# Unveiling the thermal and aqueous stability of 1D lepidocrocite titania

**DOI:** 10.1039/d6na00382f

**Published:** 2026-08-20

**Authors:** Risha A. Iythichanda, Sukanya Maity, Mustafa Mahmoud Aboulsaad, Tomas Edvinsson, Johanna Rosen, Per O.Å. Persson

**Affiliations:** a Department of Physics, Chemistry and Biology (IFM), Linköping University 58183 Linköping Sweden per.persson@liu.se risha.achaiah.iythichanda@liu.se sukanya.maity@liu.se; b Wallenberg Initiative Materials Science for Sustainability (WISE), Linköping University, Department of Physics, Chemistry and Biology (IFM) 58183 Linköping Sweden johanna.rosen@liu.se; c Department of Materials Science and Engineering, Solid State Physics, Uppsala University 75105 Uppsala Sweden mustafa.aboulsaad@angstrom.uu.se tomas.edvinsson@angstrom.uu.se

## Abstract

One-dimensional (1D) lepidocrocite titanium dioxide (TiO_2_) filaments are investigated with respect to their thermal and aqueous stability. Structural and phase evolution are examined by *in situ* heating in vacuum using (scanning) transmission electron microscopy combined with electron energy loss spectroscopy and under ambient conditions using Raman spectroscopy. The filaments retain their lepidocrocite structure up to ∼300 °C, above which localized sintering and amorphization occur at filament overlap junctions. With further heating, the amorphous regions crystallize into anatase TiO_2_, with Raman spectroscopy corroborating the onset of structural disorder. Long-term aqueous storage (>100 days) under ambient conditions induces transformation into flake-like anatase nanoparticles. This process is strongly suppressed under refrigerated storage, where no structural changes are observed over the same period. These results establish critical thermal and environmental stability thresholds that define operational advantages and limits for emerging applications of 1D lepidocrocite TiO_2_ filaments.

## Abstract 2

1D lepidocrocite TiO_2_ filaments remain structurally stable up to 300 °C in vacuum, but prolonged heating or extended aqueous exposure induces transformation to anatase. Refrigeration significantly slows the phase transformation.

## Graphical abstract

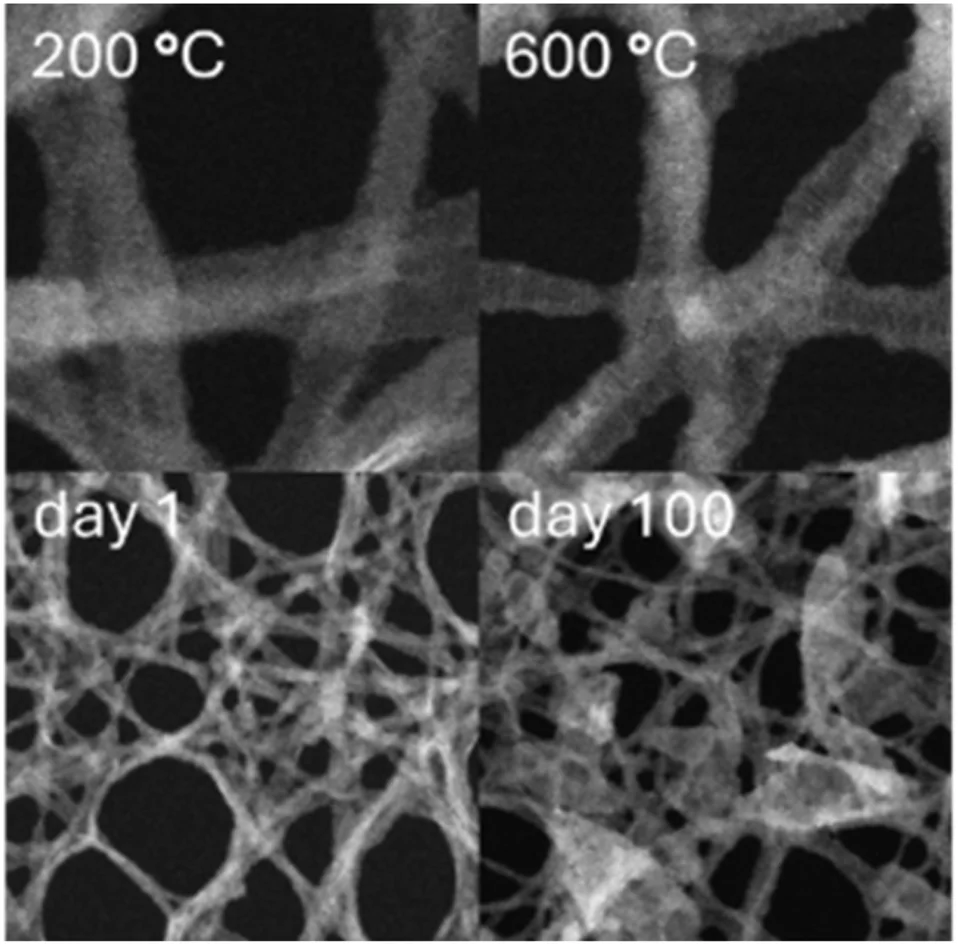

## Introduction

Over the past few decades, low-dimensional materials (LDMs) such as two-dimensional (2D)^1^ and, more recently, one-dimensional (1D) materials^2^ have attracted significant attention in materials science. The reduced dimensionalities give rise to pronounced changes in fundamental properties like the quantum confinement effect,^3^ structural anisotropy,^4^ photon transportation,^5^ and mechanical flexibility^6^ and offer a new platform for tuning electronic,^7^ optical^8^ and catalytic properties.^9^ 1D nanostructures exhibit a unique opportunity as a consequence of their further enhanced aspect ratio,^10^ leading to staggering gravimetric and volumetric surface area,^11^ which renders them highly attractive for applications in, *e.g.*, energy storage,^12^ catalysis,^9,13^ optoelectronics,^14^ water purification,^15^ and biomedical systems.^16^ These opportunities make LDMs prime candidates for next generation functional materials targeting the UN global goals.^17^

1D lepidocrocite structure titanium dioxide (filaments)^18^ stand out among the recently discovered LDMs due to its exceptional structural properties. The dimensions of these filaments comprise lengths beyond 1 µm, a width of typically 3–6 nm, and a thickness of a single unit cell. These ribbons only grow in a single direction and are crystalline throughout the filament. The filaments bend significantly and exhibit a high amount of single atom vacancies, such as Ti- and O- vacancies. Furthermore, the edges of the filaments exhibit a disordered structure.^19^ Their geometry thus results in an exceptional surface to volume ratio, a cotton-like material with high permeability, a large bandgap, and tunable electronic characteristics. While TiO_2_ in its anatase and rutile forms has been extensively studied for a range of applications,^20^ much less is known about these filaments in terms of fundamental properties. In terms of applications, they are still largely unexplored. However, the filaments exhibit a unique combination of properties, including high material permeability with exceptionally large surface area. These characteristics make them promising materials for application in ambient environments. Such features establish these materials as strong candidates for future applications and contribute to the development of more efficient, sustainable and scalable technologies.^18^

Previous reports have largely focused on the synthesis, morphology, and immediate properties of these filaments, with thus far limited attention given to their stability, despite their novelty and prospects. While TiO_2_ in other forms has been widely studied in terms of stability and phase transitions,^21,22^ there remains a gap in the understanding of stability of filaments under both thermal and environmental conditions. This is of crucial practical concern, as their performance in real world applications depends on their ability to retain structural integrity over time.

This report presents a systematic investigation of the thermal stability and phase evolution of these filaments in a vacuum, focusing on their behavior under controlled heating (up to 600 °C), as well as their stability during prolonged storage under ambient and refrigerated conditions. By performing (scanning) transmission electron microscopy (TEM) combined with electron energy loss spectroscopy (EELS), by *in situ* heating, in a vacuum, and Raman spectroscopy under ambient conditions, we provide comprehensive insights into the thermal stability. Further X-ray photoelectron spectroscopy (XPS) analysis was carried out to verify the surface chemical composition. Beyond this we also explore the stability of the filaments during extended storage under aqueous conditions. Accordingly, this study fills an important gap in the understanding of the filaments and provides boundary conditions for their practical application throughout various fields.

## Results

The thermal stability of the filaments in a vacuum was investigated using *in situ* heating (S)TEM. 1D lepidocrocite TiO_2_ filaments were stored in aqueous solution and drop cast on a MEMS heating chip. Accordingly, deposited filaments are randomly organized and overlap between two or more filaments occurs spontaneously. This can be seen in the STEM images in Fig. 1, where the mass-thickness dependent contrast results in brighter regions where filaments overlap, compared to regions with single filaments or vacuum (dark). To avoid contamination by species from the microscope vacuum, the samples were first heated to, and imaged, at 100 °C, at which point the filaments exhibit their pristine morphology, see Fig. S1. Upon heating to 200 °C, the filaments retain their structural integrity as demonstrated in Fig. 1A and D. The overview image shows an intricate network of filaments, while the high magnification image shows single filaments with homogeneous contrast (constant thickness and mass), see label 1, Fig. 1D and S1. In regions where filaments overlap, the boundaries can be clearly seen and contrast varies with integer steps (dependent on the number of filaments projected in the direction of the e-beam), see label 2 in Fig. 1D and S1.

**Fig. 1:**
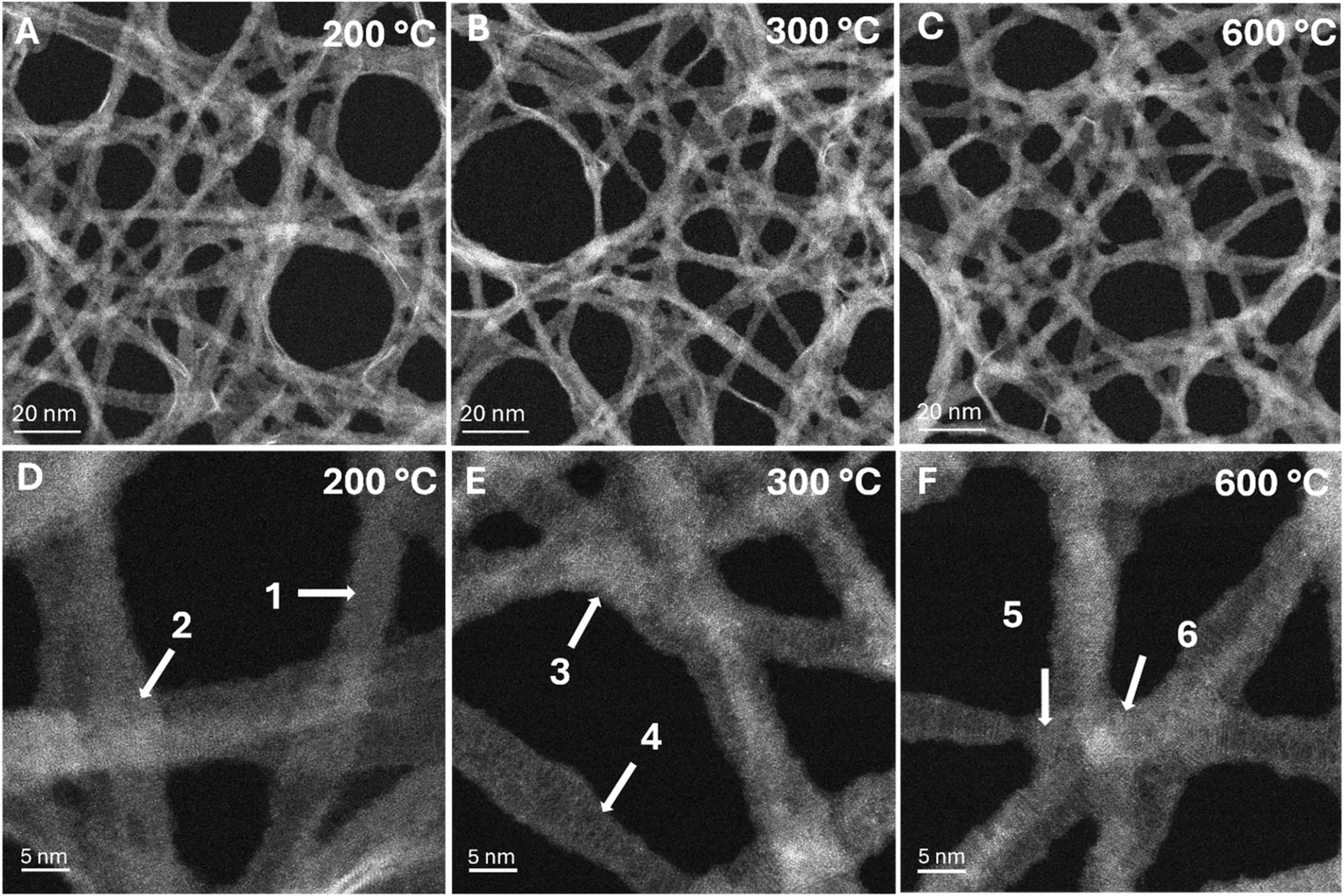
Overview and high magnification STEM images of the *in situ* thermal stability experiment at 200 °C (A and D), 300 °C (C and F), and 600 °C (B and E), respectively.

Upon heating to 300 °C, the overview image in Fig. 1B shows no distinct changes. However, upon closer inspection of the high magnification image in Fig. 1E and S1 initial changes in the overlapping regions become apparent. The close contact between two or more filaments results in a localized increase in thickness, accompanied by structural disorder at the edges. Finally, it is no longer possible to distinguish one filament from another, indicating signs of local sintering (label 3, Fig. 1E and S1). In overlapping regions, the sintering results in localized crystalline disorder. In contrast, the single filaments remain largely unaffected, and the lepidocrocite phase is preserved (label 4, Fig. 1E and S1).

With the further increase of annealing temperature to 400 °C and 500 °C (Fig. S2(b–e) respectively), a further pronounced structural transformation is evident. The lepidocrocite phase persists in the non-overlapping regions (label 1, Fig. S2d), while sintering and amorphization continue in regions where filaments overlap (label 2, Fig. S2d). Notably, at 500 °C the amorphous regions begin to re-crystallize (label 4, Fig. S2e), whereas the single filaments (label 3, Fig. S2e) display local atomic defects (vacancies) that disrupt the regular atomic arrangement in the filaments and can also be seen to form local vacancy clusters.

After heating to 600 °C significant morphological and structural changes occur to the point that even the overview image exhibits a duller appearance compared to the overview images at lower temperatures. From the high magnification image in Fig. 1F and S1f, it can be observed that the overlapping regions of the filaments show pronounced recrystallization, where the amorphous phase has transformed into anatase (label 6, Fig. 1F and S1f). Simultaneously, the single filaments begin to degrade. This can be seen *e.g.*, by necking of the filaments (label 5, Fig. 1F and S1f) and by an enhanced contrast near the filament edges which is indicative of an amorphous material that exhibits a higher thickness compared to the otherwise unit cell thick filament. Together these are indications of thermal breakdown with a projected conversion from the lepidocrocite phase into anatase TiO_2_. The filaments were investigated in parallel by electron energy loss spectroscopy (EELS) throughout the temperature range, providing insights into changes in the local chemistry. Spectra were acquired by averaging information across a filament rich region, as extended exposure to the high energy electrons in localized regions otherwise affects the structure of the filaments. Fig. 2 follows the Ti L_3,2_ and O K-edges throughout the heating process. The spectra remain largely unchanged from 200 to 500 °C, showing no significant variation in the fine structure, peak positions, or relative intensities. This spectral stability is consistent with the structural observations made using STEM. The lack of any distinct spectral shifts reinforces the conclusion that the lepidocrocite phase remains stable up to 500 °C in regions without overlap. However, at 600 °C, both the Ti L_3,2_ and the O K-edges exhibit notable differences with respect to the lower temperatures. For the Ti L_3,2_ edge, this is most prominently visible by the emerging separation of the t_2g_ and e_g_ crystal field splitting features within both the L_2_ and L_3_ edges, accompanied by a small but noticeable ∼0.25 eV chemical shift towards higher energy of the edge features. The edge thus assumes the characteristic profile of anatase TiO_2_, which enhances the interpretation of crystallization of the amorphous phase. Also, the O K-edge at 600 °C reveals the appearance of a distinct hybridization feature and a more pronounced chemical shift, associated with Ti 3d and O 2p orbital interactions which are also typical for the anatase structure.

**Fig. 2:**
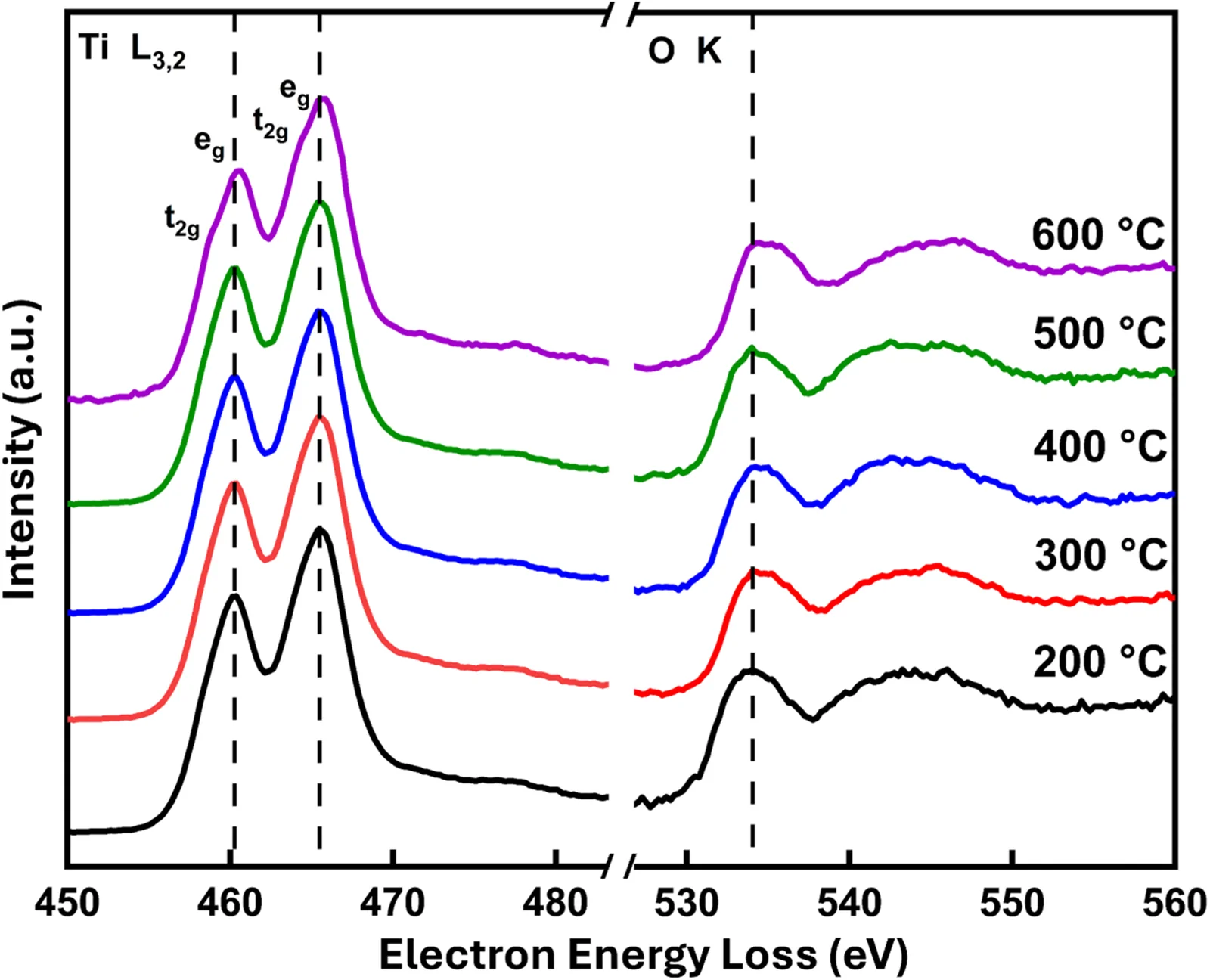
EELS core-loss spectra of the Ti L_3,2_ and O K-edges of the filaments after heating to temperatures ranging from 200 to 600 °C.

To exclude electron beam induced artefacts and confirm that the observed changes arise from thermal effects, we performed *in situ* Raman spectroscopy from room temperature (RT) up to 300 °C, see Fig. 3, following the same heating protocol used during the *in situ* STEM experiment. Unlike STEM, the Raman measurements were conducted under ambient atmospheric conditions rather than in a vacuum. At RT, 11 characteristic Raman peaks are observed at 115, 189, 275, 288, 388, 449, 699, 755, 841, 920, and 951 cm^−1^, consistent with layered lepidocrocite titanates.^22^ The bands in the 600–940 cm^−1^ region are assigned to Ti–O vibrations.^23^ In particular, three Ag modes at 269, 434, and 824 cm^−1^ indicate a well-developed layered lepidocrocite framework.^24^ The absence of the *E*_g_ mode at 144 cm^−1^, characteristic of anatase TiO_2_, further corroborates the lepidocrocite phase purity.^25^

**Fig. 3:**
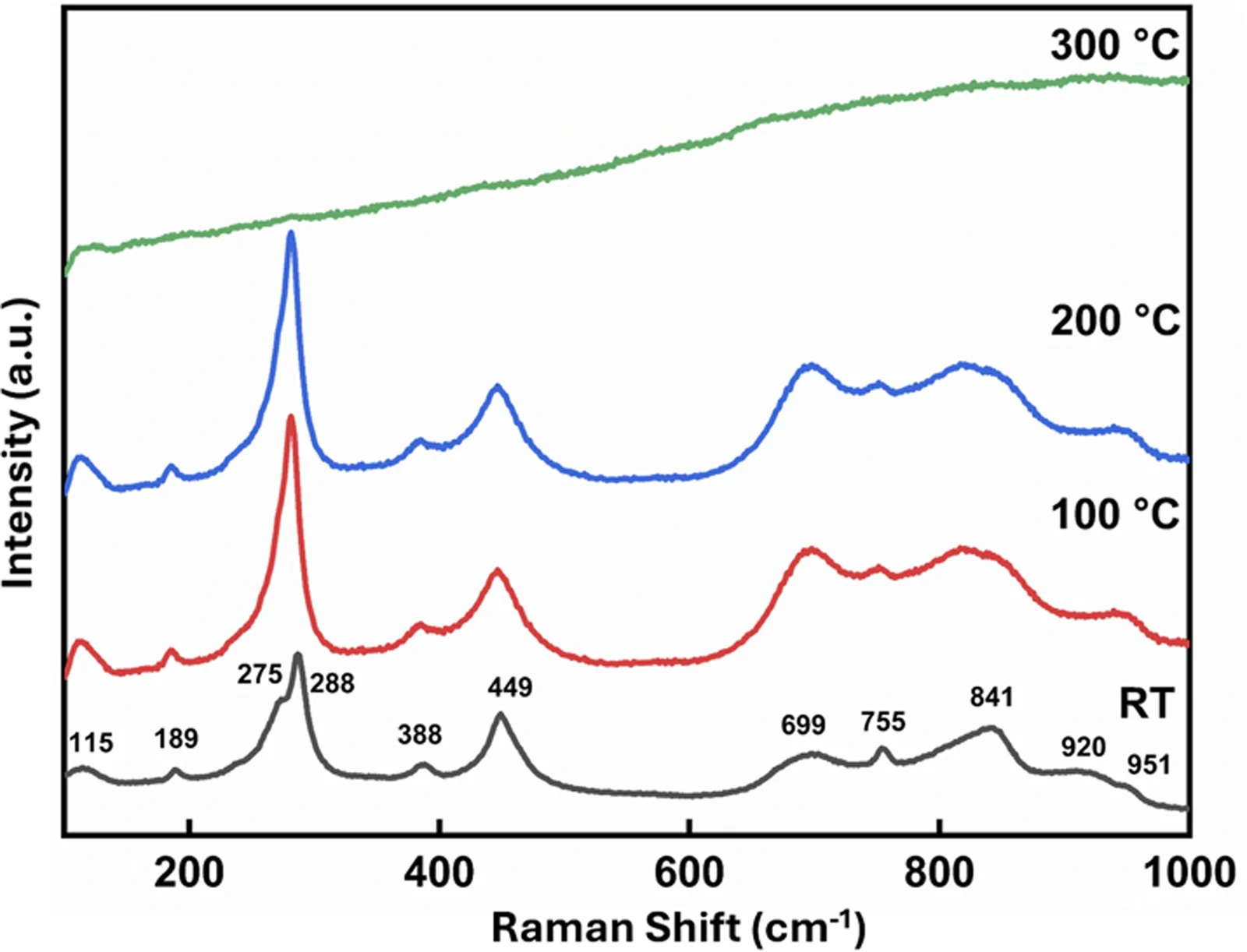
*In situ* Raman spectra of 1D lepidocrocite titania at different temperatures.

Moreover, the lack of a 665 cm^−1^ Raman band confirms the absence of the 2D lepidocrocite TiO_2_ phase,^26^ as this feature originates from the ∼700 cm^−1^ Ag band when 2D lepidocrocite TiO_2_ co-exists with 1D lepidocrocite TiO_2_. With increasing temperature, the spectra remain characteristic of lepidocrocite TiO_2_ and show no significant evolution. The diagnostic peaks of orthorhombic lepidocrocite at 187, 280, 382, 446, 799, and 951 cm^−1^ persist throughout the entire temperature range.^24,27^ At 300 °C, however, the spectra are dominated by a broad fluorescent background. This is consistent with our prior *in situ* heating STEM observations, wherein overlapping regions began to sinter at 300 °C. For the *in situ* Raman measurements, the specimen was prepared by drop-casting multiple filament layers onto a glass substrate, producing extensive overlap. Upon heating to 300 °C, these overlapping regions interact and generate amorphous regions across the network, which in turn yield the observed fluorescence. Collectively, these Raman data support the conclusion that the structural responses are thermally driven rather than beam induced. Furthermore, to verify the surface chemistry, XPS analysis was carried out at room temperature on the pristine sample and the corresponding spectra are provided in Fig. S3. The obtained spectra are consistent with previously reported results for the same material^12^ and for anatase TiO_2_.^32^

Following the thermal stability experiments, the filaments were dispersed in a colloidal solution (4 mg ml^−1^) and stored under ambient and refrigerated conditions in the dark to monitor their long-term stability. As shown in Fig. 4A, the filaments exhibited structural stability during the first week. However, with increasing storage duration, the gradual formation of flakes was observed. As shown in Fig. 4B, showing the filaments after 100 days of storage, the presence of flake-like particles becomes increasingly evident, and after 150 days, as shown in Fig. 4C, the colloidal solution is densely populated with flakes, as further illustrated in the overview images in Fig. S4a and b. The flakes were identified as single crystal TiO_2_ anatase particles, as demonstrated by high resolution scanning transmission electron microscopy (HRSTEM) analysis, see Fig. S5. Statistical analysis of TEM images yielded an average in the plane aspect ratio (length/width) of 2.36 ± 0.53 (*n* = 38). For the flakes where the thickness could be determined, the average length to thickness aspect ratio was 8.33 ± 2.62 (*n* = 30), confirming the anisotropic morphology of the TiO_2_ nanoflakes.

**Fig. 4:**
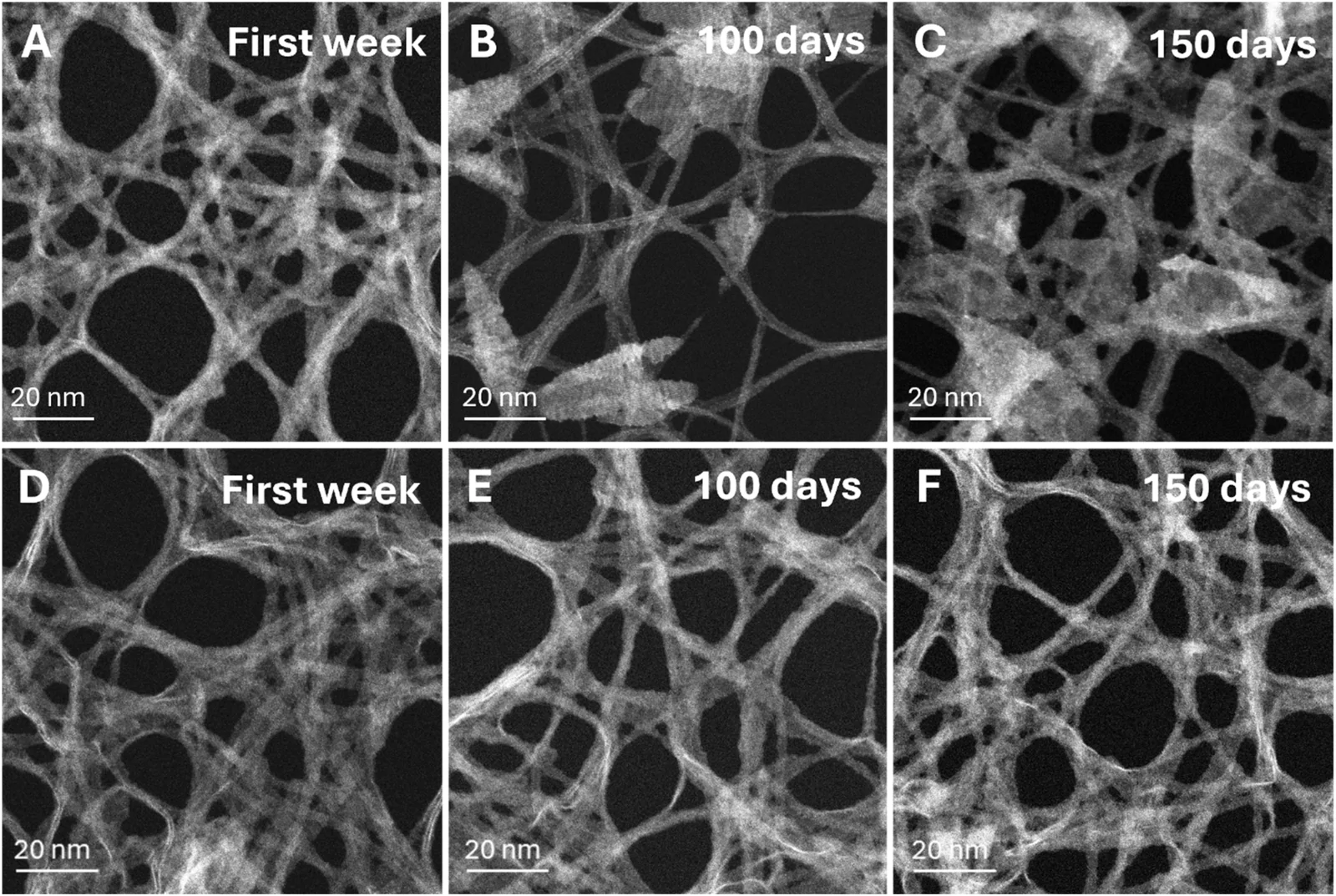
Aqueous stability of 1D lepidocrocite titania stored under ambient (A–C) and refrigerated conditions (D–F), imaged at 200 °C.

Also, for these samples, EELS was employed. At the early stages of the experiments, Ti L_3,2_ edge spectra are characteristic of the lepidocrocite phase, see Fig. S6. However, by 100 days, significant changes emerge in the spectral features, particularly the appearance of the distinct t_2g_ peaks, which are clear signatures of anatase formation. Additional confirmation is provided by the oxygen K edge, see Fig. S6. Around the 100-day mark, distinct hybridization features develop, reflecting a transformation in the local oxygen coordination environment into anatase TiO_2_ and signifying the progressive conversion of lepidocrocite filaments over time.

In parallel, an identical colloidal solution (4 mg ml^−1^) was stored under refrigerated conditions (4 °C) and monitored over the same period (Fig. 4D–F). In this case no formation of competitive phases or degradation of the filaments was observed throughout the period, indicating that the reduced temperature significantly suppresses the transformation process. This finding clearly underscores that increased temperature or environmental conditions facilitate the degradation and phase transition.

Notably, the high synthesis yield (∼98–99%) ensures that nearly all precursor material is converted into lepidocrocite and that there are negligible unreacted materials or byproducts available to account for the formation of the anatase particles. Accordingly, the appearance of flakes can be inferred to occur as the result of degradation of the filaments under ambient conditions, due to an intrinsic instability and transformation of the filaments over time. Interestingly, despite the internal transformation, no visible changes in the physical appearance (*e.g.*, color, turbidity or precipitation) of the colloidal solution were observed during the 150-day period (Fig. S7a and b) emphasizing the need for microscopic and/or spectroscopic techniques to detect the changes.

## Discussion

The thermal evolution of lepidocrocite TiO_2_ filaments under *in situ* heating reveals a complex interplay of structural stability, defect formation, and phase transformation, which depends on filament morphology and arrangement. Notably, the sintering observed in the overlapping regions is presumably a consequence of the system's tendency to minimize its total surface and interfacial energy at elevated temperatures. When two or more filaments are in close contact, the localized increase in thickness, combined with the observed disorder at the edges of the filaments, promotes atomic diffusion, enabled by the applied thermal energy. In contrast, the non-overlapping filaments demonstrate stability up to ∼500 °C. The absence of contact between filaments removes the driving force for sintering. However, these regions are not unaffected as structural defects begin to emerge in the filament core as the temperature increases. These defects, most likely involving titanium and oxygen vacancies, are presumably thermally activated point defects, likely to form at elevated temperature in the (S)TEM vacuum. The formation of vacancies and vacancy clusters weakens the crystal lattice and is a well-documented behavior in TiO_2_.^28,29^ As vacancies accumulate, they increase the probability for lattice disruption and lead to increasing crystalline disorder that eventually results in structural breakdown, even in the absence of overlap between filaments.

The most significant structural transformation occurs at 600 °C, where sintered amorphous regions of the filaments undergo recrystallization into the anatase phase. This transition aligns with known behavior of titania materials under thermal treatment, while the formation is not simply due to thermal melting, but a thermally driven phase transition.^30^ During this process, the atomic mobility increases, especially at higher temperatures, allowing atoms to shift into a more stable phase.^31^ In non-overlapping regions at 600 °C, the emerging defects increase the crystalline disorder that eventually leads to structural breakdown, with fractures and lattice misalignments becoming more apparent.

When *in situ* Raman spectroscopy was performed the filaments indicated structural stability up to 200 °C in air. However, the Raman signals become obscured by a competing photoluminescence background as early as 300 °C onwards, in contrast to TEM (which shows complete structural degradation at 600 °C). This can be rationalized by differences in sensitivity of the technique but also partly by the sample preparation and resulting filament density in the samples. In the *in situ* Raman experiment, the sample was prepared by drop casting multiple layers of the filaments, resulting in a denser film, consequently exhibiting more overlap compared to the sample prepared for TEM. The broad fluorescent background in the Raman spectra at 300 °C is indicative of the start of the structural breakdown occurring at the same temperature as for the initial observations of sintering in the TEM. Therefore, the results are not contradictory but rather reflect the sensitivity of the technique to bulk and surface defect formation in a denser and more thermally responsive configuration. The difference in the atmosphere (ambient air in Raman spectroscopy *vs.* vacuum in TEM), spatial averaging (Raman probes a micrometer scale area *vs.* an atomic scale in TEM), and sample architecture collectively explain the spectral changes observed in Raman spectra.

The revealed thermal behavior of lepidocrocite TiO_2_ filaments provides key insights into the potential applications and limitations. A thermal stability up to 300 °C suggests suitability for applications such as photocatalysis, low temperature sensors, energy storage components, and transparent coating in optoelectronics. Maintaining phase purity and structural integrity in this range is crucial for optical, electronic, and catalytic performance. The formation of the anatase in the overlapping region after 300 °C can motivate future investigations into the functional performance of the interconnected anatase network, particularly where high surface area and efficient charge transport are critical. Tailoring the filament connectivity and nanoscale morphology may provide a pathway towards enhanced material performance. However, the observed phase transition and structural changes at higher temperatures limit their use in higher temperature processes, in thermal cycling, or under extreme conditions.

Moving to aqueous stability, the lepidocrocite filaments remain structurally intact and colloidally robust for an extended period (∼100 days) under ambient conditions. Throughout this timeframe, the material preserves their characteristic 1D morphology and visual appearance, suggesting that the material is sufficiently stable for most practical processing and application workflows. This long stability window is particularly encouraging. Only after prolonged storage beyond ∼100 days is the structural transformation into anatase observed under ambient conditions. Notably, this transformation progresses slowly and can be effectively suppressed by storing the colloidal solution under refrigerated conditions. Overall the filament stability makes them well suited for applications that capitalize on the unique 1D morphology, such as colloidal inks, photocatalysis, biosensing, and electrochemical devices. The material performs reliably within the operational and processing timescale typical for these applications. Although exceedingly long term ambient storage eventually induces partial transformation, simple strategies such as cold storage and surface modification are sufficient to preserve structural integrity. Thus, the observed behavior does not diminish their practical utility; rather, it provides valuable guidance for handling and long-term preservation.

## Conclusion

This study provides insight into the thermal and aqueous stability of 1D lepidocrocite TiO_2_ filaments using *in situ* STEM, EELS, and *in situ* Raman spectroscopy. A film of filaments remains thermally stable below 300 °C, retaining its lepidocrocite phase and structural integrity. Sintering between overlapping filaments, resulting in amorphization, is observed in STEM from 300 °C onwards. However, individual filaments demonstrate remarkable thermal robustness and remain stable up to >500 °C. A distinct phase transformation from amorphous into anatase TiO_2_ occurs at ∼600 °C, as confirmed by both imaging and spectroscopy. In an aqueous colloidal system, the filaments remain structurally stable for the initial period under ambient conditions, but gradually transform into anatase TiO_2_ over 150 days, as evidenced by the appearance of anatase flakes, which is attributed to the intrinsic instability of the lepidocrocite phase under ambient conditions. Conversely, a sample stored under refrigerated conditions showed no signs of degradation or phase change, highlighting the critical role of temperature for long term colloidal stability. In summary, this study reveals the thermal and environmental stability and limitations for 1D lepidocrocite filaments and offers crucial insights for the application of this material.

## Experimental methods

### Sample preparation

Filaments were prepared according to previously published methods.^18^

### Materials

Titanium(iv) oxysulfate (TiOSO_4_) (≥29% wt% Ti, anhydrous basis, powder) and tetramethylammonium hydroxide (TMAH) (25 wt%) were procured commercially (Sigma Aldrich). Ethanol (EtOH) 200 proof, was obtained from VWR Chemicals.

### Experimental details

The one-pot synthesis method for creating 1D lepidocrocite TiO_2_ filaments involves mixing the TiOSO_4_ powder in the TMAH solution in a Ti : TMA molar ratio of 0.4 at temperatures of 80 °C. First, TiOSO_4_, in powder form, was added to the TMAH solution in polyethylene bottles into which a hole was poked to prevent any pressure buildup. The system was kept on a magnetic stirrer in an oil bath for 2 days. When TiOSO_4_ dissolved in TMAH a clear solution was obtained. After the reaction, a white sediment was obtained that was in turn washed with EtOH several times until the pH was ≈7 to remove the excess TMA and then centrifuged until the supernatant was clear. After discarding the supernatant, the precipitate was dispersed in DI water and shaken until a clear, transparent colloid was formed. This colloid solution is further used for characterization.

### Sample preparation for *in situ* STEM and *in situ* Raman spectroscopy

The filaments for the *in situ* STEM investigation were drop cast from an aqueous dispersion on SiN_*x*_ MEMS heating chips. The MEMS chip was mounted on a DENSsolutions holder and inserted into the TEM, where the sample was heated at 100 °C overnight in a vacuum environment to eliminate any potential contamination. The thermal stability of these filaments was studied up to 600 °C, with a temperature interval of 100 °C and a duration of 15 minutes at each temperature step. After each heating step, the sample was returned to 100 °C for imaging and EELS analysis. For Raman spectroscopy, a thin film of the filaments was prepared by drop casting ∼5 mg of material on a glass substrate. The long-term storage experiments were conducted by placing colloidal solutions (4 mg ml^−1^) of filaments under ambient and refrigerated conditions. In the experiment the initial procedure is the same as that of the thermal stability test but here all the imaging and EELS analysis were carried out at 200 °C.

### Sample characterization

Scanning TEM (STEM) high-angle annular dark-field (STEM-HAADF) and EELS were performed using a double-corrected FEI Titan^3^ 60–300, operated at 300 kV. For the *in situ* thermal characterization, a MEMS based heating chip (DENSsolutions) was mounted in a DENSsolutions Wildfire heating holder. The chip consists of an electron transparent SiN membrane with an integrated resistive microheater. To enhance imaging quality, a focused ion beam (FIB) system (Thermo Scientific Helios 5 UC DualBeam) was employed to prepare holes in the SiN windows, improving electron transparency and resolution. The heating holder ensured stable electrical contact with the MEMS device and enables the observation of structural and morphological evolution at different temperatures, while minimizing thermal drift and maintaining high spatial resolution.

Raman spectra were acquired using a Renishaw inVia Reflex confocal microscope, equipped with a frequency doubled Nd:YAG laser operating at 532 nm with a maximum power of 43 mW at the sample surface. The single spot and mapping spectra were collected using a 50× long working distance objective (NA 0.5), a 2400 lines per mm grating, and a laser power adjusted to 10% of the maximum power (4.3 mW) using neutral density filters to avoid any deterioration from the laser heating. The spectra were recorded with 20 seconds of integration time and 5 accumulations, covering a static scan range of 85–1345 cm^−1^. Prior to spectral acquisition of the samples, the spectrometer was calibrated with measurements on silicon, confirming that the characteristic Si peak is found at 520.5 cm^−1^.

The XPS were obtained by using an Axis Ultra DLD instrument from Kratos Analytical. The incident X-rays were monochromatized Al Kα radiation (*h* = 1486.6 eV). The base pressure in the system during spectral acquisition is 1.1 × 10^−9^ Torr. and the anode power is set to 150W. All spectra are collected from an area of 0.3 × 0.7 mm^2^ and at the normal emission angle. As the material is a semiconductor, a charge neutralizer was not used. The material is not etched before analysis as the film is so thin and it can damage the film.

## Author contributions

Risha Achaiah Iythichanda: conceptualization, investigation, visualization, writing – original draft. Sukanya Maity: investigation. Mustafa Mahmoud Aboulsaad: investigation, writing – original draft. Tomas Edvinsson: writing – review and editing, supervision. Johanna Rosen: writing – review and editing, supervision. Per O. Å. Persson: conceptualization, writing – review and editing, supervision. All authors reviewed the manuscript, contributed to the discussion and interpretation of the results, revised the manuscript, and approved the final version.

## Conflicts of interest

The authors declare no conflicts of interest.

## Supplementary Material

NA-008-D6NA00382F-s001

## Data Availability

The authors confirm that the data supporting the findings of this work are available within the article and supplementary information (SI). The raw data are also accessible from the corresponding author upon reasonable request. Supplementary information: overview and high magnification STEM images of the *in situ* thermal stability experiment at 100 °C, 400 °C and 500 °C, overview, STEM images, and HRSTEM image of anatase TiO_2_ flakes, aqueous stability of 1D lepidocrocite titania, EELS core-loss spectra, Ti L_2,3_-edge and O K-edge, XPS, and physical appearance of 1D lepidocrocite titania at room temperature (PDF). See DOI: https://doi.org/10.1039/d6na00382f.
