# Supplementary material for: Unveiling the thermal and aqueous stability of 1D lepidocrocite titania
Source: Nanoscale Adv. 2026 Aug 20;8(20):5850–7. doi: 10.1039/d6na00382f (PMC13492517; doi:10.1039/d6na00382f)
Supplement: Supplementary file 1 [file NA-008-D6NA00382F-s001.pdf]

Supplementary Information (SI) for Nanoscale Advances.
This journal is © The Royal Society of Chemistry 2026

Supporting information:

Unveiling the Thermal and Aqueous Stability of 1D Lepidocrocite Titania

Risha Achaiah Iythichanda [a,b], *Sukanya Maity* [a], *Mustafa Mahmoud Aboulsaad* [c], *Tomas Edvinsson* [c], *Johanna Rosen* [a,b], *and Per O.Å. Persson* *, [a,b]

[a] Department of Physics, Chemistry and Biology (IFM), Linköping University, 58183 Linköping, Sweden

[b] Wallenberg Initiative Materials Science for Sustainability (WISE), Linköping University, Department of Physics, Chemistry and Biology (IFM), 58183 Linköping, Sweden

[c] Department of Materials Science and Engineering; Solid State Physics, Uppsala University, 75105 Uppsala, Sweden

* Corresponding Author

E-mail address: per.persson@liu.se (Per O.Å. Persson)

Figure S1. High magnification STEM images of the in situ thermal stability experiment from Figure1 (d), (e), (f)

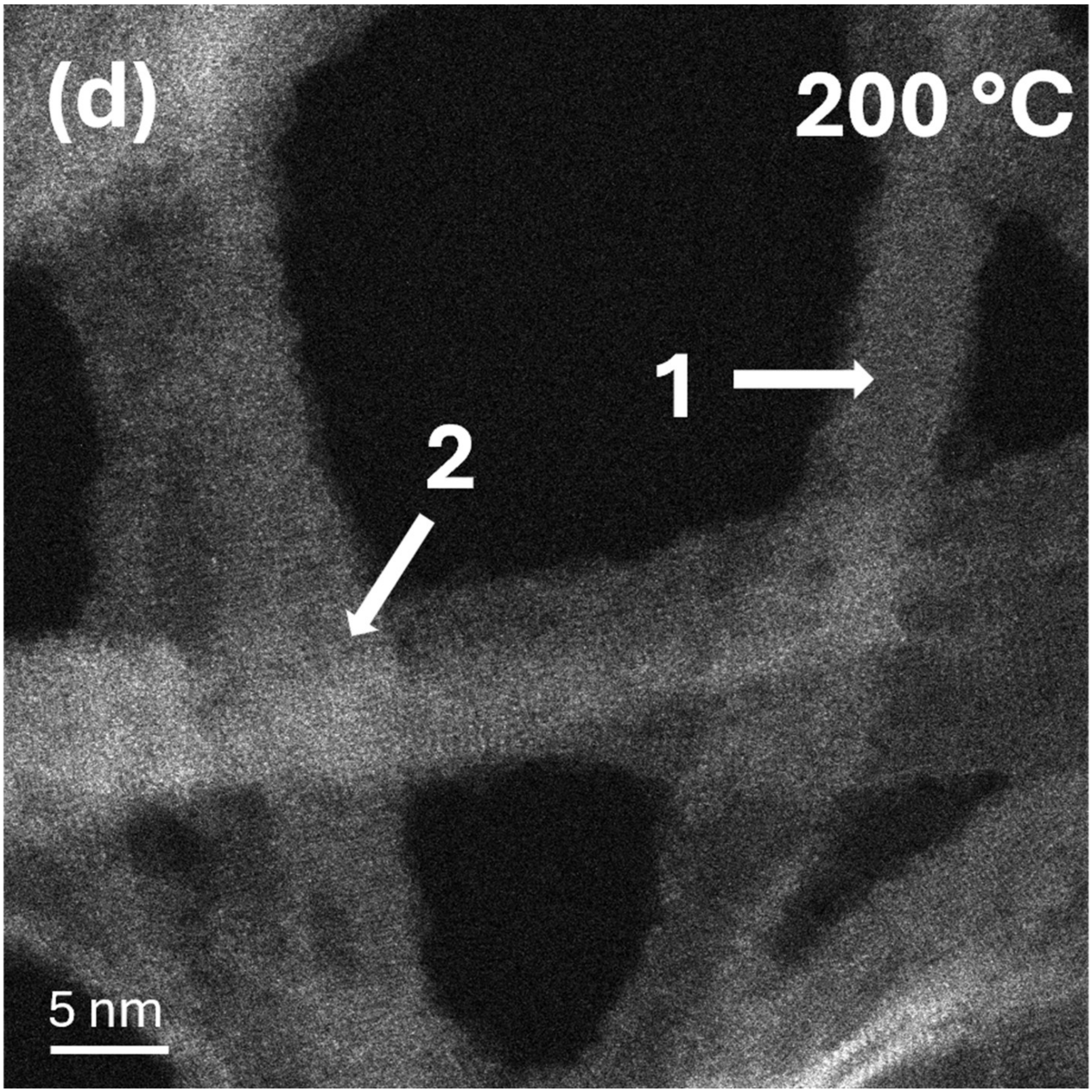

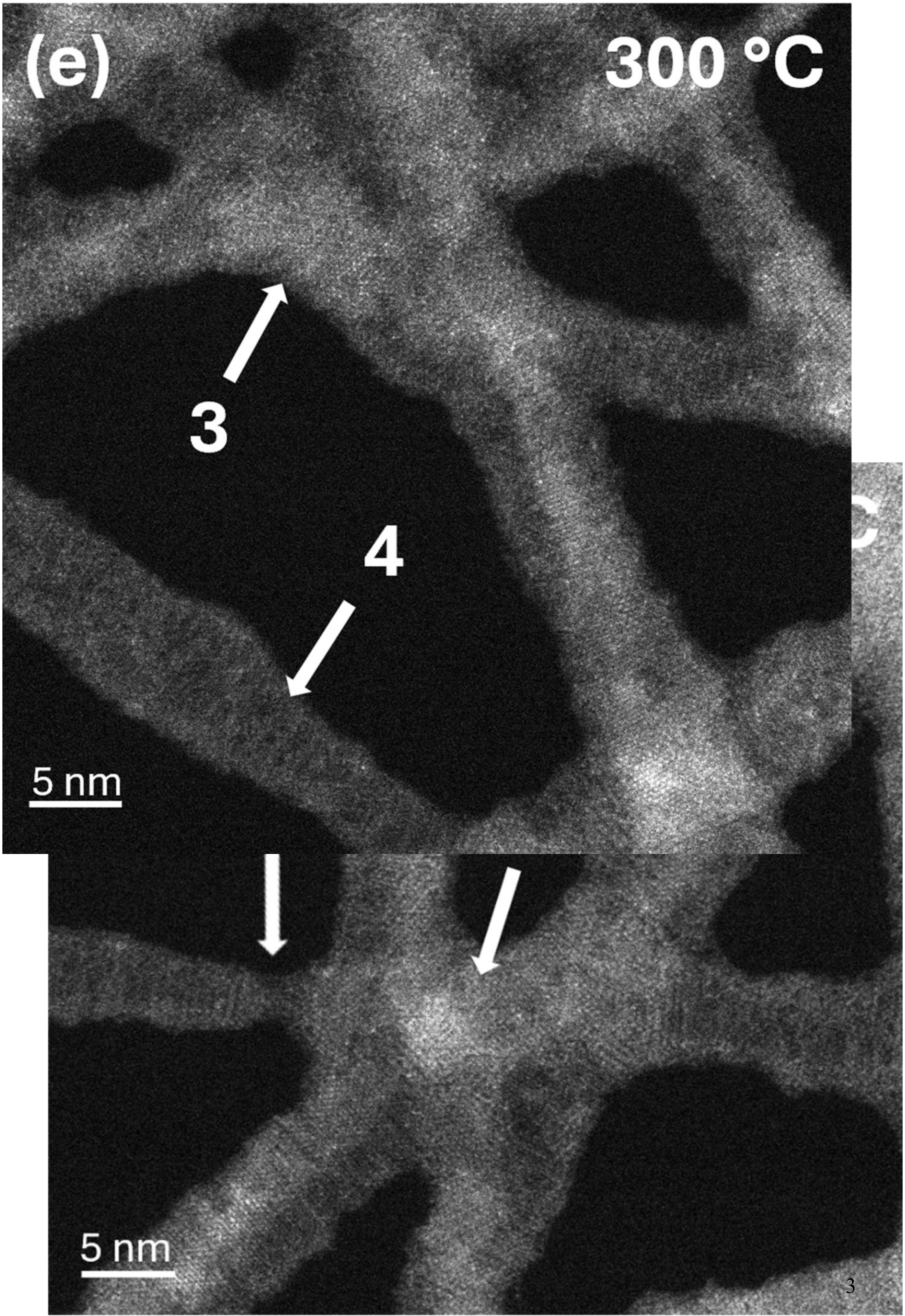
(e)
300 °C
3
4
5 nm
5 nm

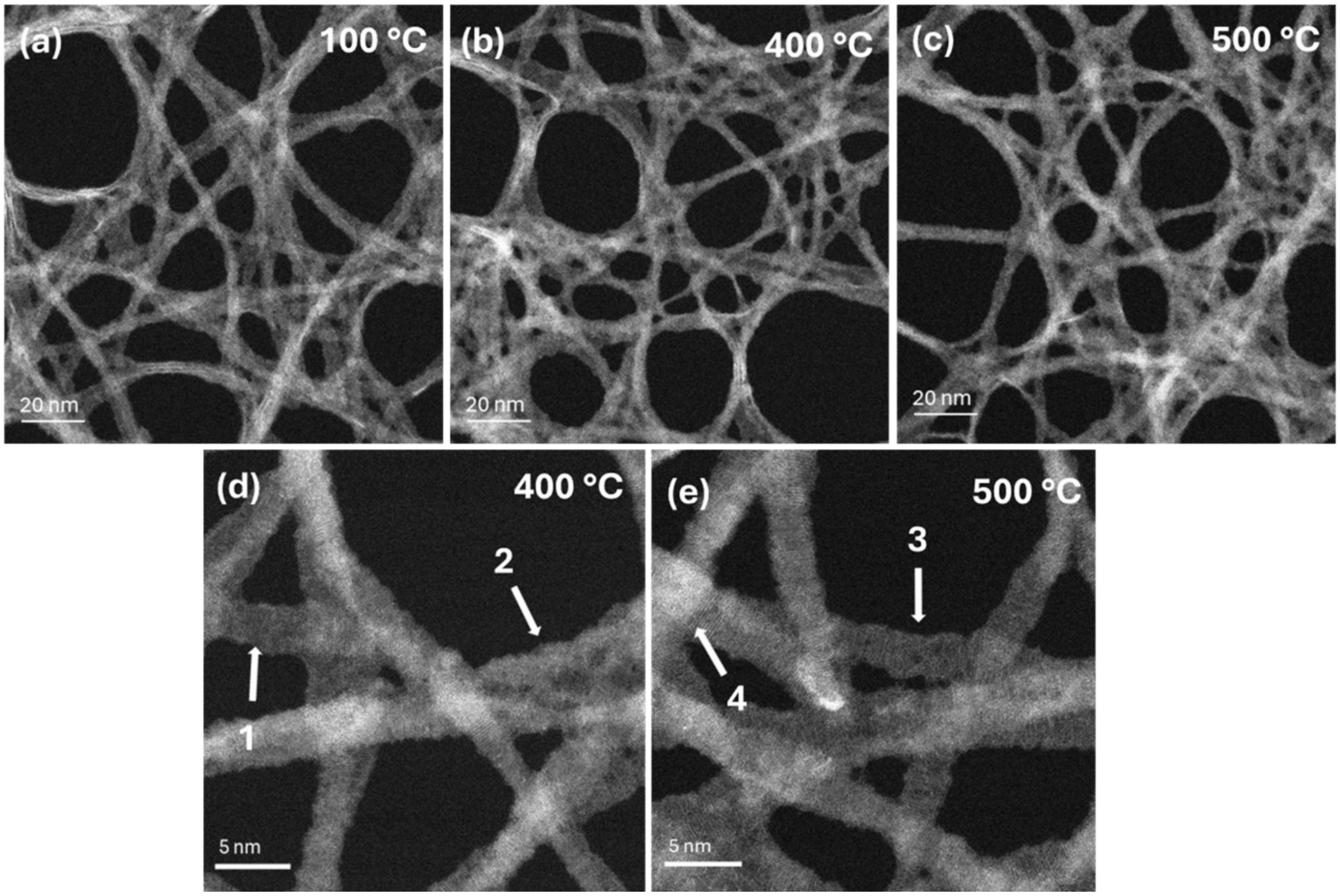

Figure S2. Overview and high magnification STEM images of the in situ thermal stability experiment at 100 °C (a), 400 °C (b, d), and 500 °C (c, e), respectively.

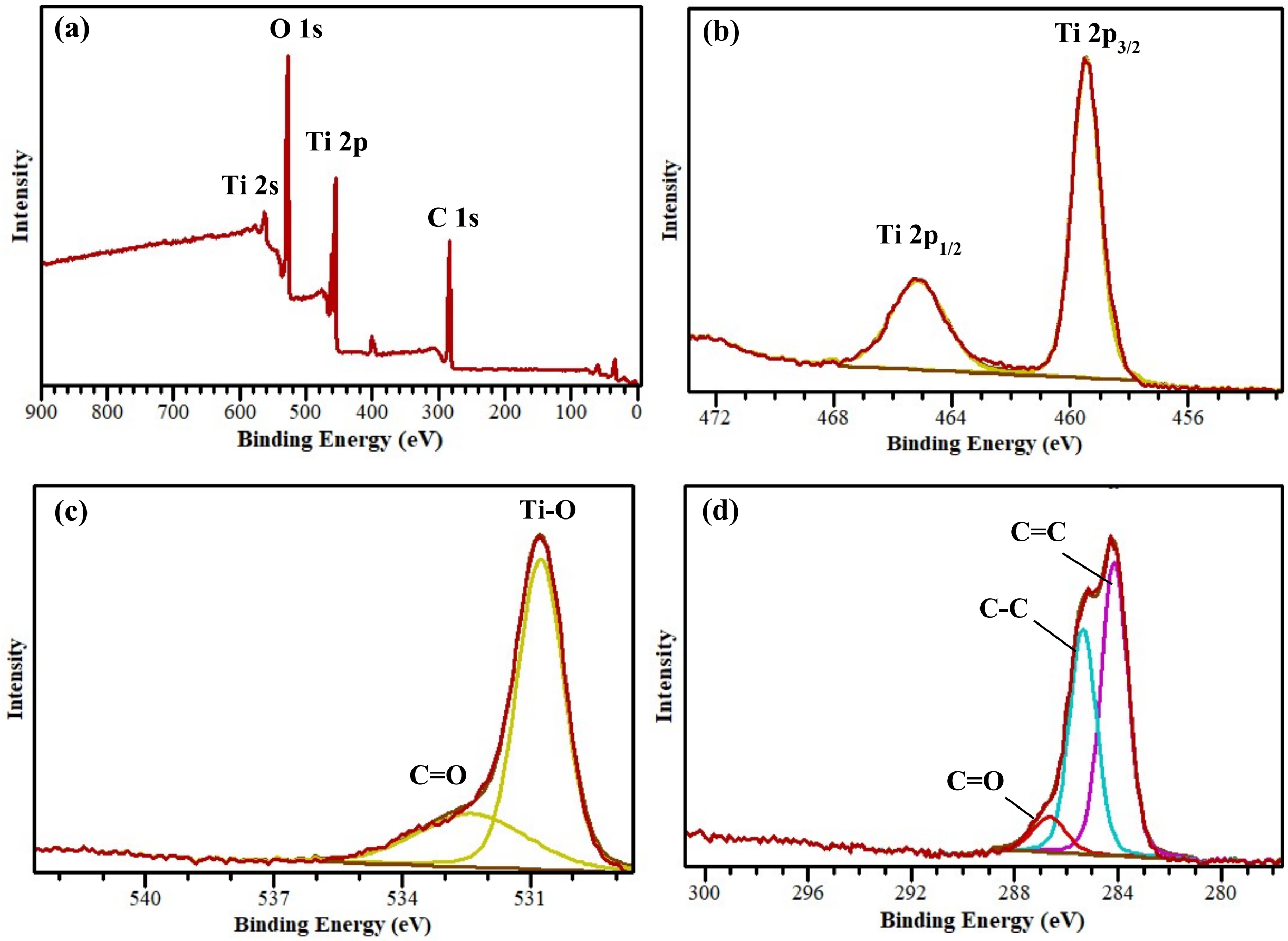

Figure S3. XPS spectra (a) Survey scan, (b) Ti 2p spectra, (c) O 1s spectra, (d) C 1s spectra

The XPS survey spectrum (Fig. 1a) confirms the presence of Ti and O in the synthesized material. The high-resolution Ti 2p spectrum (Fig. 1b) shows two prominent peaks at binding energies of 459.5 eV and 465.2 eV, corresponding to Ti $2p_{3/2}$ and Ti $2p_{1/2}$, respectively. These values are characteristic of Ti^{4+} in TiO_2. The spin orbit splitting of 5.7 eV further confirms the formation of TiO_2. The O 1s spectrum (Fig. 1c) can be deconvoluted into two components located at ~530 eV and ~532.6 eV, attributed to Ti–O bonds and surface C=O species, respectively. The dominant intensity of the Ti–O peak indicates the formation of TiO_2, while the C=O contribution is associated with minor surface carbon contamination.[1,2]

The high-resolution C 1s spectrum (Fig. 1d) shows peaks at ~284.5 eV and ~285.37 eV, corresponding to C–C/C=C bonds. These features indicate the presence of residual carbon species and oxygenated functional groups, such as hydroxyl or epoxy groups, likely arising from surface

adsorption or processing conditions. These results collectively confirm the expected chemical states and surface composition of the synthesized TiO_2.[1]

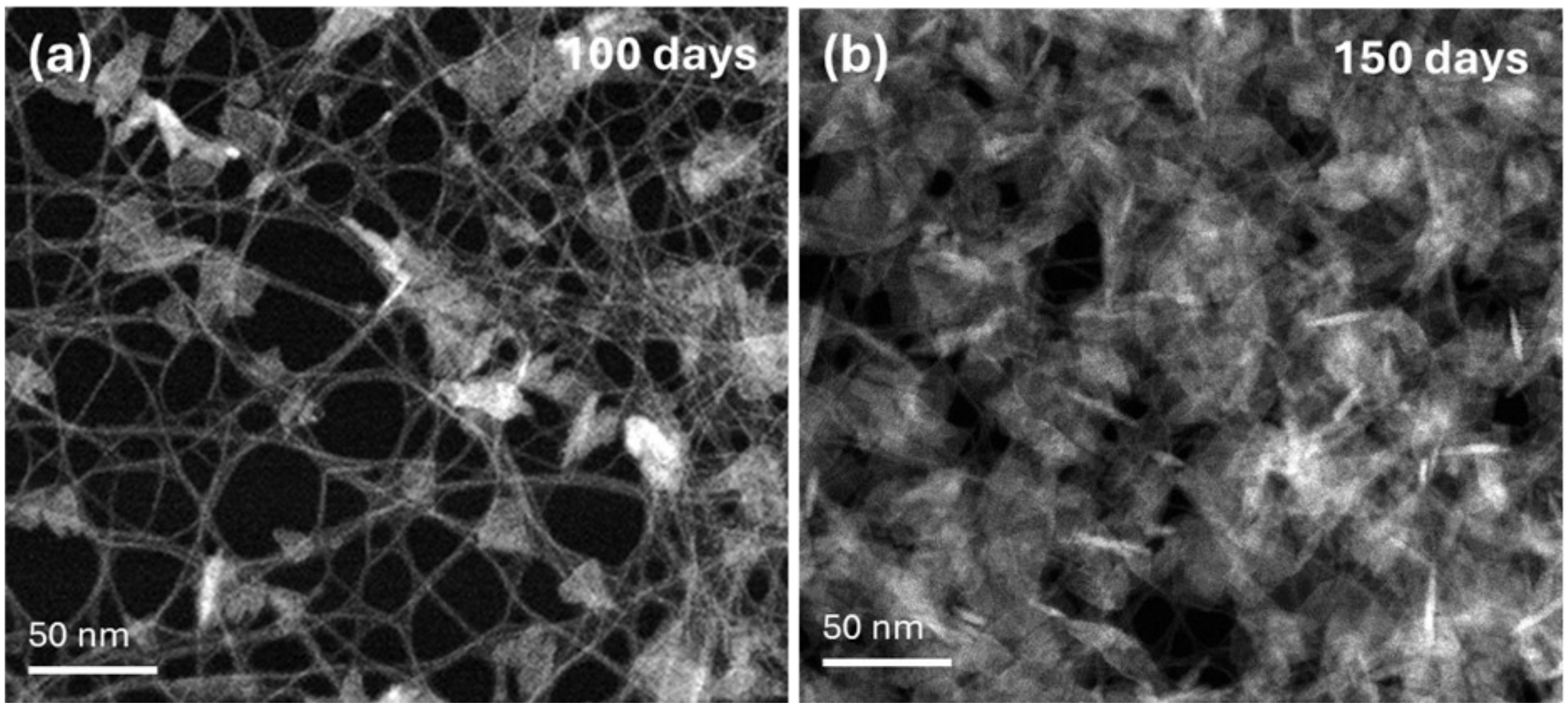

Figure S4. Aqueous Stability of 1D Lepidocrocite Titania: Overview

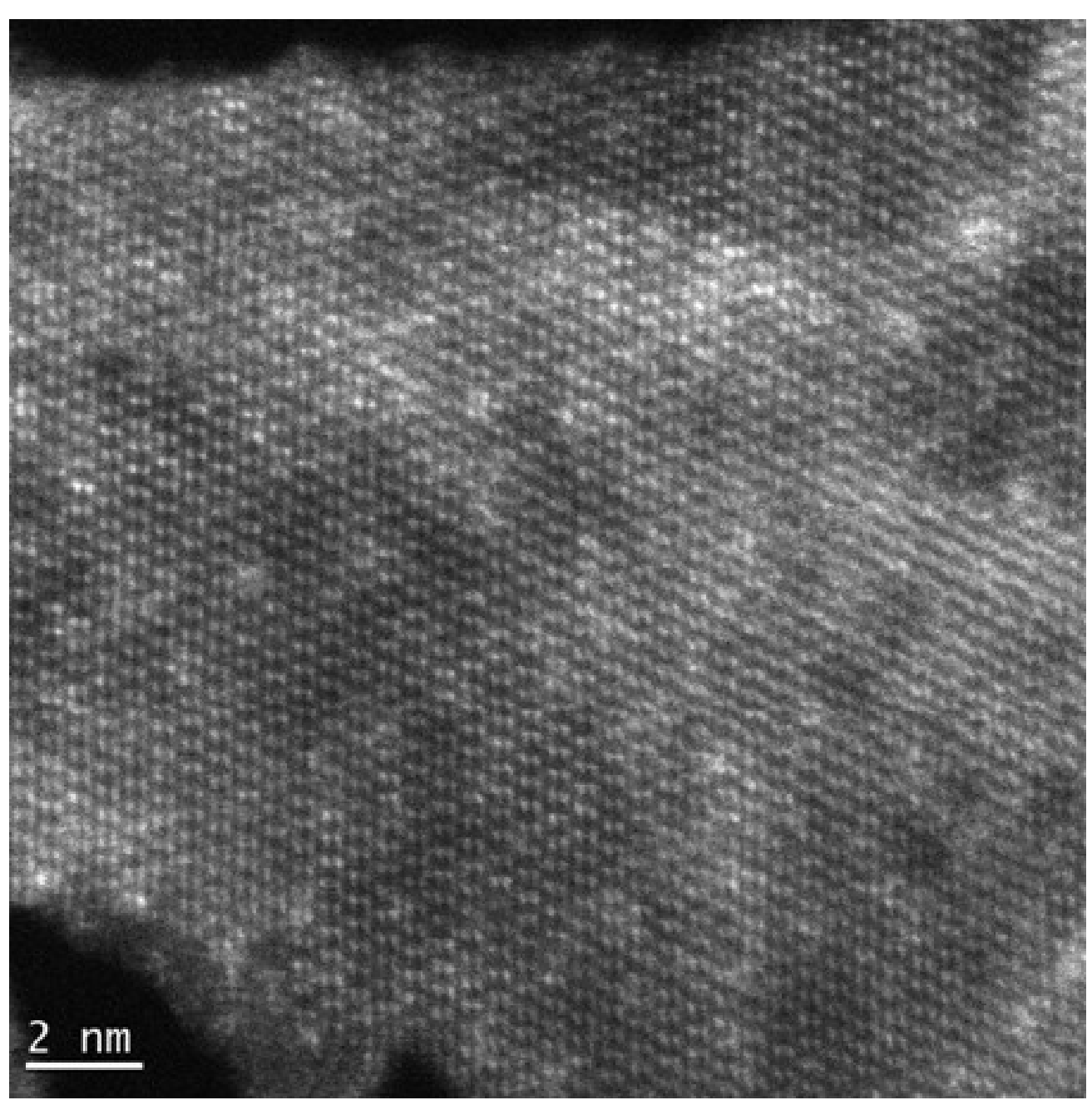

Figure S5. HRSTEM image of anatase TiO_2 flake

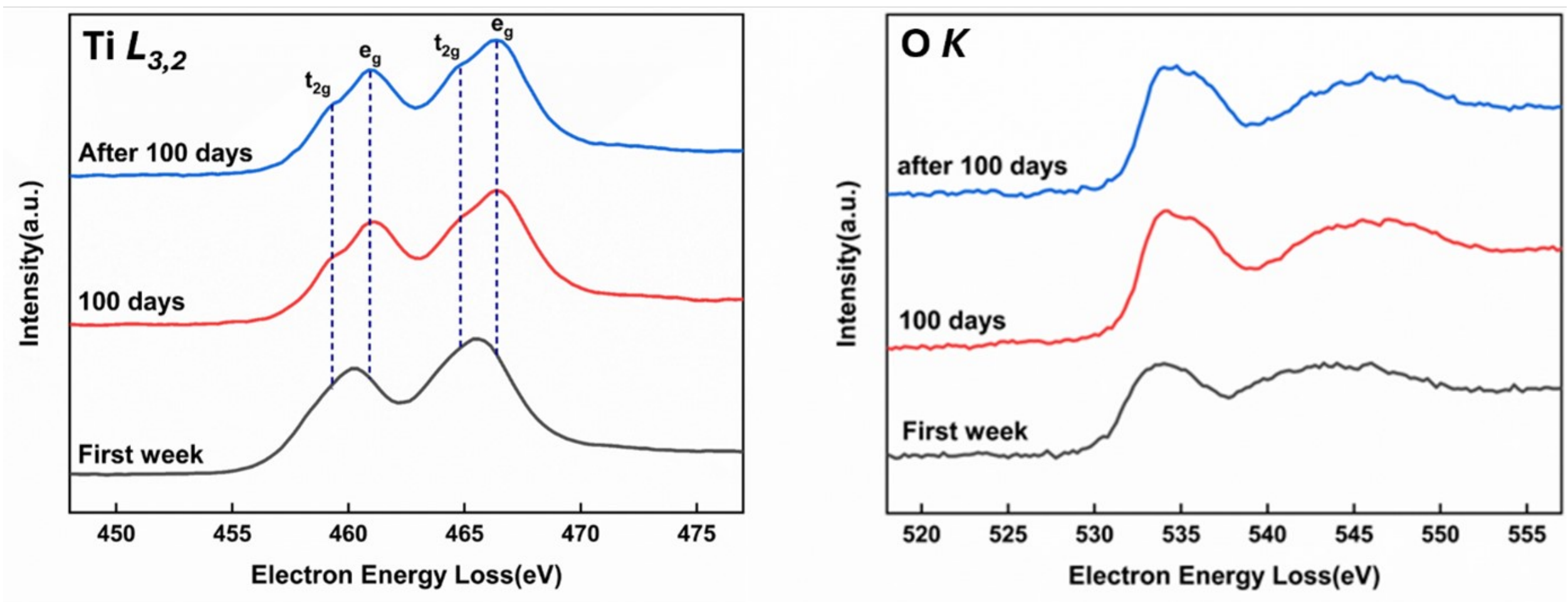

Figure S6. Aqueous stability of 1D Lepidocrocite Titania EELS core-loss spectra Ti $L_{2,3}$-edge and O K-edge

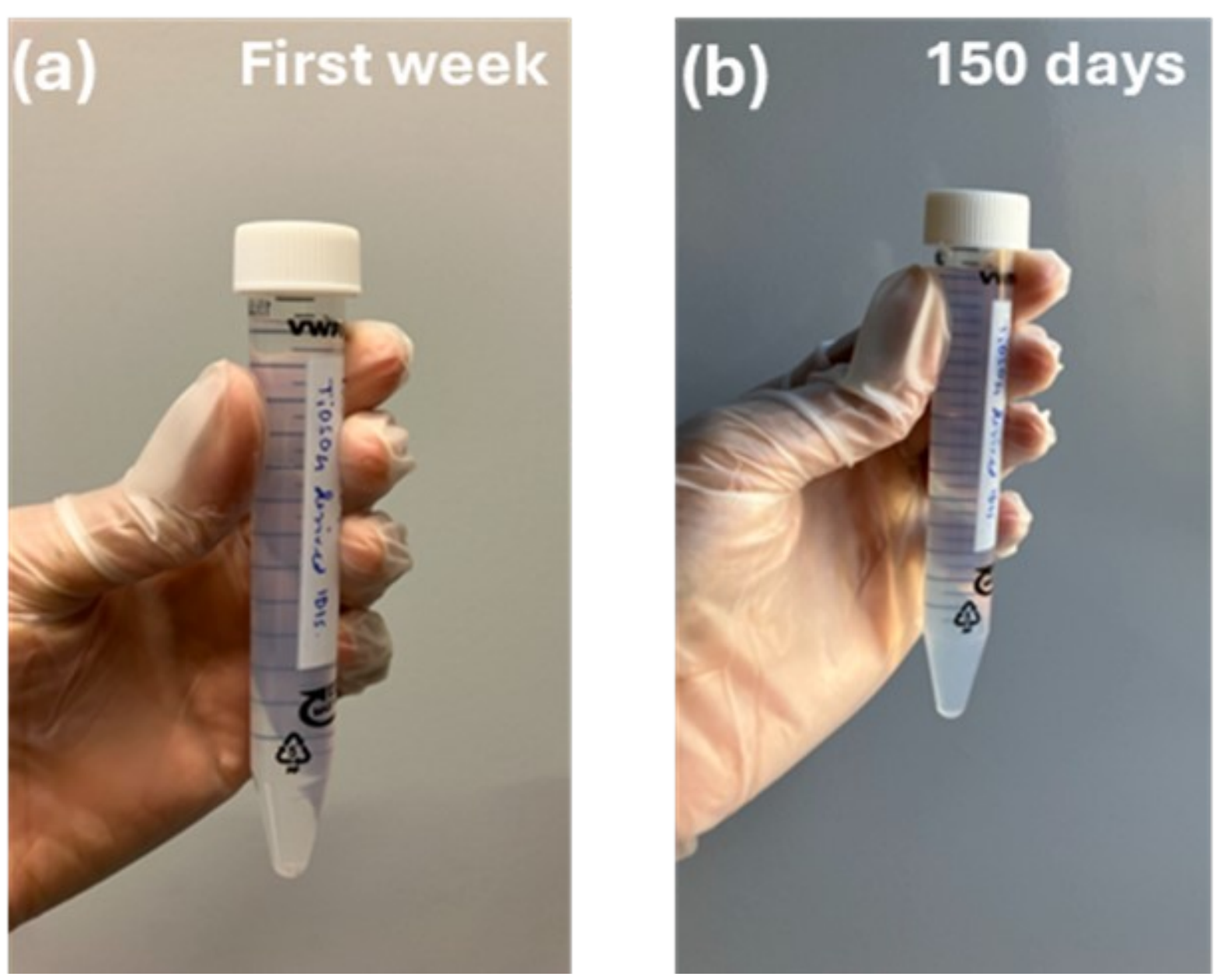

Figure S7. Physical appearance of 1D lepidocrocite Titania in room temperature

Reference

(1) Maity, S.; Qin, L.; Petruhins, A.; Barsoum, M. W.; Rosén, J. One-Dimensional Lepidocrocite Titania Mesoparticles Integrated with Activated Carbon for High-Performance Supercapacitor Applications. *J. Energy Storage* **2024**, *101*, 113895.

(2) Herman, G. S.; Gao, Y.; Tran, T. T.; Osterwalder, J. X-ray Photoelectron Diffraction Study of an Anatase Thin Film: TiO_2(001). *Surf. Sci.* **2000**, *447* (1–3), 201–211.
